# Enhanced Catalytic Activity of TiO_2_/rGO Nanocomposites with Squaraine Dye for Photocatalytic Wastewater Treatment

**DOI:** 10.3390/ma19194102

**Published:** 2026-09-25

**Authors:** Petya Karakashkova, Maya Shopska, Iliyana Yordanova, Georgi Tyuliev, Georgi B. Hadjichristov, Stela Minkovska

**Affiliations:** 1 Institute of Catalysis, Bulgarian Academy of Sciences, Acad. G. Bonchev Str., Bl.11, BG-1113 Sofia, Bulgaria; shopska@ic.bas.bg (M.S.); yordanova@ic.bas.bg (I.Y.); tyuliev@ic.bas.bg (G.T.); 2 Georgi Nadjakov Institute of Solid-State Physics, Bulgarian Academy of Sciences, 72 Tzarigradsko Chaussee Blvd., BG-1784 Sofia, Bulgaria; georgibh@issp.bas.bg; 3 National Centre of Excellence—Mechatronics and Clean Technologies, 8 Kliment Ohridski Blvd., Blk. 8, BG-1756 Sofia, Bulgaria

**Keywords:** titanium dioxide/graphene oxide, squaraine dye, photocatalytic degradation, laser dyes

## Abstract

Wastewater treatment has stimulated researchers to develop highly efficient catalysts for the degradation of organic pollutants. The present work focuses on the design and investigation of a titanium dioxide (TiO_2_)-based photocatalytic system with improved catalytic ability under visible light. TiO_2_/reduced graphene oxide (TiO_2_/rGO) and squaraine dye (SQ) sensitized TiO_2_/rGO (TiO_2_/rGO/SQ) composites were synthesized by a facile solvothermal method. The incorporation of SQ aims to enhance the photocatalytic efficiency of the synthesized composites and extend their activity by utilizing the visible-light spectrum. The composite materials were characterized by X-ray photoelectron spectroscopy (XPS), Fourier transform infrared spectroscopy (FT-IR), scanning electron microscopy (SEM), X-ray diffraction analysis (XRD), the Brunauer–Emmett–Teller (BET) surface area analysis, and UV–Vis diffuse reflectance spectroscopy (DRS). The synthesized composites were evaluated for the decolorization of the organic dyes Rhodamine B (RhB) and Fluorescein (FL) in aqueous solutions. The synthesized composites were evaluated for the decolorization of the organic dyes Rhodamine B (RhB) and fluorescein (FL) in aqueous solution. Under visible-light irradiation, TiO_2_/rGO/SQ achieved 98% decolorization of FL and 86% decolorization of RhB within 180 min.

## 1. Introduction

Over the last few decades, photocatalysis and photocatalytic materials have become important fields of contemporary science and technology with a wide range of applications [1,2,3]. Over the last few decades, photocatalysis and photocatalytic materials have become important fields of contemporary science and technology with a wide range of applications [1,2,3]. In particular, photocatalytic processes have been extensively investigated for the degradation of organic pollutants in water, an issue of increasing importance in view of industrial and technological development [4]. Significant progress has been achieved in this area of environmental protection [5].

Among semiconductor photocatalysts, titanium dioxide (TiO_2_) has been extensively investigated for environmental applications, because of its chemical stability, low toxicity, and relatively low cost [5]. Nevertheless, its photocatalytic performance is restricted by its wide band gap (~3.2 eV) and the rapid recombination of photogenerated charge carriers, which reduce the efficiency of the photocatalytic redox processes [6,7].

Graphene-based materials have also been widely investigated as components of photocatalytic degradation of organic pollutants [8]. Graphene oxide (GO) can accept photogenerated electrons from a semiconductor, thereby prolonging charge separation and extending the lifetime of charge carriers [9]. Furthermore, the high specific surface area and favorable adsorption properties of graphene-derived materials can promote interaction between organic molecules and the photocatalytic surface, thereby contributing to improved photocatalytic performance [10]. Numerous studies on photocatalysts based on reduced graphene oxide (rGO) coupled with TiO_2_ nanoparticles demonstrated that rGO can serve as an effective co-catalyst, significantly enhancing photocatalytic performance in the degradation of various organic pollutants such as textile dyes and pharmaceutical compounds [11,12,13].

Currently, a large number of studies have focused on the modification of semiconductor photocatalytic materials. The aim is to enhance photon efficiency and the ability to utilize both the UV and visible regions of the solar light spectrum. Visible-light photocatalysis is considered an economically favorable, environmentally friendly technology for the removal of organic pollutants. The development of relevant effective photocatalytic materials is crucial for their widespread application. Various strategies have been employed to improve the activity of inorganic photocatalysts, including dye sensitization and coupling with carbon nanostructures such as fullerenes, carbon nanotubes, GO, and rGO [14,15].

Another approach for extending the activity of semiconductor photocatalysts into the visible spectral region involves the employment of organic dyes as sensitizers. With this approach, organic dyes adsorbed on the semiconductor surface absorb visible light and transfer excited electrons into the conduction band of the semiconductor, thereby extending its photoresponse into the visible region and improving photocatalytic performance [16,17]. Dye sensitization has been widely investigated for TiO_2_-based photocatalysts owing to its simplicity, low cost, and effectiveness in utilizing solar energy. Although numerous organic dyes have been investigated as sensitizers, the application of squaraine dyes in photocatalysis has received relatively limited attention, despite their strong absorption in the visible and near-infrared regions and their favorable photochemical stability [18,19,20]. Due to their highly conjugated molecular structure and excellent photochemical and thermal stability, these organic dyes have found applications in photocatalysis, organic photovoltaics, and dye-sensitized solar cells [21,22].

Previous studies have established that sensitization by squaraine dye can extend the photocatalytic activity of TiO_2_ into the visible spectral region and significantly improve its photocatalytic efficiency under solar light [17,20]. In our previous work, we reported the synthesis and photocatalytic evaluation of a ternary composite of TiO_2_/rGO and a squaraine dye, which exhibited significant activity in the decolorization of the organic dye Methylene Blue in aqueous solution under visible light irradiation [23]. In the present study, using a specially synthesized squaraine dye (hereafter abbreviated as SQ), we have prepared a TiO_2_/rGO/SQ composite by a facile solvothermal method and evaluated the photocatalytic activity of this material for the decolorization of the organic dyes Rhodamine B (RhB) and Fluorescein (FL) under visible-light irradiation. These dyes are characterized by high stability and persistence in aqueous environments; therefore, their effective degradation is essential for the environment due to their toxicity.

The squaraine dye SQ was chosen as a sensitizer owing to its molecular architecture, which is favorable for visible-light-driven photocatalysis. The highly conjugated zwitterionic π-electron system, composed of two indolenine units linked through a squaraine core, provides intense absorption in the visible region and promotes efficient intramolecular charge transfer upon photoexcitation. These structural features are expected to facilitate electron injection from photoexcited SQ into the conduction band of TiO_2_. In addition, the oxygen atoms of the squaraine core may interact with coordinately unsaturated Ti(IV) surface sites, providing electronic coupling between the dye and the semiconductor. Thus, SQ is expected to be a promising sensitizer to extend the photoresponse of TiO_2_/rGO nanocomposite photocatalyst to the visible spectral region.

## 2. Materials and Methods

All chemicals and solvents were of analytical grade and were used as received without additional purification. Tetrabutyl titanate and isopropanol were purchased from Sigma-Aldrich (Milwaukee, WI, USA), and graphene oxide was purchased from Graphenea (San Sebastian, Spain). RhB was purchased from Sigma-Aldrich (St. Louis, MO, USA), and fluorescein (FL) from Fluka AG (Buchs, SG, Switzerland).

### 2.1. Materials and Preparation of Photocatalysts

#### 2.1.1. Synthesis of the Squaraine Dye (SQ)

The squaraine dye (SQ), 3-oxo-4-[(1,3,3-trimethyl-3H-indol-1-ium-2-yl) methylene]-2-[(1,3,3-trimethylindolin-2-ylidene)methyl]cyclobut-1-enolate (Figure 1) was prepared by the previously reported microwave-assisted procedure [24] Briefly, a mixture of 1,3,3-trimethyl-2-methyleneindoline (10 mmol), squaric acid (5 mmol), and ethyl L-lactate (10 mL) were heated at 140 °C (450 W) for 5 min. The reaction mixture was subsequently cooled and transferred into ice water. The resulting solid was collected by filtration, rinsed with hot water, and allowed to dry in air. The isolated yield was 81%.

#### 2.1.2. Synthesis of TiO_2_/rGO and TiO_2_/rGO/SQ Composites

Tetrabutyl titanate, isopropanol, and graphene oxide were used as starting materials for the preparation of the photocatalyst. The TiO_2_/rGO nanocomposite material was obtained as a powder using a simple solvothermal method following the procedure in Reference [25] with further modifications. Graphene oxide was dispersed in isopropanol by sonication for 90 min at room temperature. Tetrabutyl titanate was then introduced into the dispersion, followed by stirring for 60 min. Deionized water (1 mL) was then added dropwise to the mixture under stirring, and stirring was continued for 30 min. Subsequently, 1 mL of deionized water was introduced dropwise, and the suspension was stirred for a further 30 min. The mixture was transferred to a 100 mL Teflon-lined stainless-steel autoclave where it was kept at 180 °C for 6 h. The recovered solid was washed with ethanol and subsequently dried at 60 °C for 12 h. The resulting TiO_2_/rGO composite contained 2 wt.% rGO. The TiO_2_/rGO/SQ ternary composite (powder) was prepared as follows. SQ was dissolved in 70 mL of absolute ethanol, after which 0.6 g of TiO_2_/rGO was added to the solution. The mixture was subjected to ultrasonication for 60 min, and then magnetically stirred at room temperature for an additional 2 h. The solid was subsequently separated by filtration and dried at 60 °C to constant mass. The compositional ratio of TiO_2_/rGO/SQ was 84/1.7/14.3 (w/w/w).

### 2.2. Characterizations

The physicochemical properties of the prepared catalysts were characterized using X-ray photoelectron spectroscopy (XPS), Fourier-transform infrared spectroscopy (FT-IR), Brunauer–Emmett–Teller (BET) surface area analysis, scanning electron microscopy (SEM), powder X-ray diffraction (XRD), and UV–Vis diffuse reflectance spectroscopy (DRS).

Textural properties were evaluated by N_2_ adsorption at 77.4 K using a Quantachrome Nova 1200e analyzer (Quantachrome Instruments, Boynton Beach, FL, USA). The specific surface area was determined according to the Brunauer–Emmett–Teller (BET) method, whereas the pore-size distribution was calculated from the desorption branch of the isotherm using the Barrett–Joyner–Halenda (BJH) method. The total pore volume (Vt) and average pore diameter (Dav) were estimated according to the Gurvich rule at a relative pressure of p/p_0_ ≈ 0.99. FT-IR measurements were carried out in transmission mode using a Nicolet 6700 spectrometer (Thermo Fisher Scientific, Madison, WI, USA) over the 400–4000 cm^−1^ range. The samples were incorporated into KBr pellets at an analyte content of 0.5 wt.%. The spectra were obtained by averaging 100 scans at a resolution of 4 (data interval 1.928 cm^−1^).

XPS analysis was performed with an ESCALAB Mk II spectrometer (VG Scientific Ltd., Manchester, UK) using Al Kα radiation (hν = 1486.6 eV). Measurements were conducted at a base pressure of 10^−8^ Pa. Binding energies were referenced to the adventitious carbon C 1s signal at 284.8 eV.

SEM observations were performed with a JSM-6390 microscope (JEOL, Tokyo, Japan) using secondary electron detection (SED). The microscope was operated at an accelerating voltage of 5 kV and a working distance of 10.1 mm.

Powder XRD measurements were performed on a Bruker D8 Advance diffractometer using Cu Kα radiation and a LynxEye detector (Bruker AXS Advanced X-ray Solutions GmbH, Billerica, MA, USA). Diffraction patterns were acquired between 10° and 90° in 2θ, using a step size of 0.03° and a counting time of 17 s per step for primary phase identification. Crystalline phase composition was identified by comparison with the ICDD-PDF2 (2014).

UV–Vis diffuse reflectance spectra (DRS) were recorded using a Thermo Scientific Evolution 300 UV–Vis spectrophotometer (Thermo Fisher Scientific, Madison, WI, USA) equipped with a diffuse reflectance accessory. BaSO_4_ was used as the reference material. The spectra were recorded in the wavelength range of 350–800 nm. The diffuse reflectance data were converted to the Kubelka–Munk function, F(R), according to (F(R) = (1 − R)^2^/2R), where R is the diffuse reflectance expressed as a fraction.

### 2.3. Photocatalytic Experiments

The photocatalytic performance of TiO_2_/rGO and TiO_2_/rGO/SQ toward decolorization of representative organic pollutants, the organic dyes Rhodamine B (RhB) and Fluorescein (FL) was evaluated. Their wide industrial applications in dye lasers, textile dyeing, and the chemical and medical industries make them target compounds for evaluating the performance of photocatalytic systems. Both dyes are environmentally relevant because of their persistence and possible toxicity, which may cause adverse effects on aquatic ecosystems and human health. The photocatalytic discoloration of RhB and FL was studied in a cylindrical photocatalytic reactor with an initial reaction mixture volume of 400 mL. The reactor was equipped with a mercury lamp, Philips HPL-N, 400 W (Philips, Eindhoven, The Netherlands) combined with a 400 nm cut-off glass filter (Schott AG, Mainz, Germany) to ensure visible-light irradiation (λ > 400 nm). The light source was placed at a distance of 15 cm from the solution surface. The reaction mixture contained 0.1 g of the catalyst powder and an aqueous solution of either RhB or FL (10 mg/L, pH 7). A constant temperature of 22 °C in the reaction vessel was maintained using an external cooler. Prior to the photocatalytic experiment, 0.1 g of the catalyst was ultrasonically dispersed in water for 30 min and then transferred to the reactor under magnetic stirring. Before irradiation, the suspension was stirred in the dark for 30 min to establish adsorption–desorption equilibrium. During irradiation, O_2_ was continuously supplied to the reaction mixture at a flow rate of 6 L h^−1^. Samples of the dye solution were collected at regular time intervals during both the dark period and the irradiation period. The degree of photocatalytic decolorization of RhB and FL in aqueous solution was assessed using a UV–Vis spectrophotometer Camspec M501 (Spectronic Camspec Ltd., Leeds, UK).

## 3. Results and Discussion

### 3.1. Catalyst Characterization

As is known, the specific surface area of the catalysts plays a key role in their adsorption capacity and therefore in their efficiency. In particular, for TiO_2_- and GO-based photocatalysts, this has been proven by numerous studies on the photocatalytic degradation of water contaminants (e.g., [26,27,28,29]). The results of the BET surface area measurements performed for the synthesized TiO_2_/rGO and TiO_2_/rGO/SQ composites are presented in Table 1.

Both composites show type IV isotherms with H3 hysteresis loops in the relative pressure range of 0.4–1.0 (IUPAC classification) (Figure 2a,b), indicating the presence of mesoporous structures with a relatively narrow pore size distribution. The BET surface area of TiO_2_/rGO was 118 m^2^/g, with a total pore volume of 0.35 cm^3^/g and an average pore diameter of 12 nm. The specific surface area and pore volume of TiO_2_/rGO/SQ decreased to 106 m^2^/g and 0.31 cm^3^/g, respectively, while the average pore size remained at 12 nm.

The lower specific surface area and pore volume are consistent with the partial occupancy of the mesopore channels by SQ molecules, suggesting that the dye loading occurs within the pore network rather than being confined to the external surface [30,31]. The unchanged average pore size upon inclusion of SQ suggests that sensitization does not disrupt the mesoporous structure of the composite.

FT-IR spectra of TiO_2_/rGO and TiO_2_/rGO/SQ composites are shown in Figure 3a and Figure 3b, respectively. The broad band around 3425 cm^−1^ in both spectra corresponds to O–H stretching vibration, and the peak at 1631 cm^−1^ is due to the water –OH bending vibration, both resulting from rGO [32]. The presence of surface hydroxyl groups enhances the oxidizing capacity of the photocatalyst material, as they act as trapping sites for photogenerated holes, facilitating the formation of highly reactive hydroxyl radicals (•OH) during the photocatalytic reaction [33].

The strong and broad band in the low-frequency region in Figure 3a is assigned to stretching vibrations of Ti–O at ~534 cm^−1^ and Ti-O-Ti bonds at ~555 cm^−1^ [31], which shows formation of the TiO_2_ lattice framework within the nanocomposite [32]. The same spectral features are also observed in the spectrum of the TiO_2_/rGO/SQ composite (Figure 3b). The shoulder observed below 1000 cm^−1^ (Figure 3a) is attributed to Ti-O-C stretching vibrations, suggesting a chemical interaction between TiO_2_ and rGO, and consequently the formation of the TiO_2_/rGO nanocomposite [33]. Several absorption bands in the region of 1020–1232 cm^−1^ can be assigned to the C-O and C-O-C stretching vibrations of epoxy and alkoxy functional groups, associated with the rGO vibrational signatures [34,35]. The absence of observable spectral features near 1720 cm^−1^, corresponding to the C=O stretching vibrations of the carboxyl groups, indicates the successful reduction of GO to rGO [36].

The FT-IR spectrum of TiO_2_/rGO/SQ powder composite (Figure 3b) displays distinct absorption peaks (narrow vibrational bands) that are characteristic of the SQ dye (Figure 3c), confirming the successful incorporation of SQ molecules in the TiO_2_/rGO material. The relatively weak IR vibrational bands in the 2850–2960 cm^−1^ region can be attributed to symmetric and asymmetric C–H stretching vibrations of the methyl groups in the indolenine units of SQ [37]. The band observed at 1282 cm^−1^ in the spectra shown in (Figure 3b,c) is associated with C-N stretching vibrations of the SQ molecule. The bands observed at 1462 cm^−1^ and 1502 cm^−1^ correspond to the C=C stretching vibrations involving the four-membered squaraine ring and the aromatic heterocycle moiety of SQ. These assignments agree with previously reported data [22] and confirm the presence of SQ molecules in the TiO_2_/rGO composite. Most likely, they effectively attach to the surface of the TiO_2_ and rGO in the TiO_2_/rGO nanocomposite. Comparing the IR spectra in Figure 3, it can be observed that the addition of SQ to the TiO_2_/rGO nanocomposite does not lead to specific changes in the spectral signatures associated with both TiO_2_/rGO and SQ; i.e., no chemical bonds were formed between SQ molecules and the inorganic part of the TiO_2_/rGO/SQ composite. Most likely, the SQ molecules were attached/anchored to the TiO_2_ and rGO surfaces.

Some information about the chemical composition and bonding states of the studied TiO_2_/rGO/SQ photocatalytic composite material was obtained by XPS. In the case of graphene oxide (Figure 4a), the high-resolution C1s spectrum was deconvoluted into four peaks centered at 284.9 eV, 286.6 eV, 287 eV, and 288.9 eV, which can be attributed to C=C/C-C bonds of the aromatic framework, C-O (epoxy and alkoxy), C=O, and O-C=O, respectively [38,39]. In contrast, the C 1s spectrum of TiO_2_/rGO/SQ composite (Figure 4b) shows only three peaks centered at 284.7 eV, 286 eV, and 288.7 eV, corresponding to the C=C/C-C, C-O, and C=O groups, respectively [40]. The decrease in the intensity of oxygen-containing components, together with the disappearance of the COOH component centered at 288.9 eV, suggests that GO is largely reduced to rGO during the synthesis process (solvothermal treatment) [41]. This was confirmed by the results from IR vibrational spectroscopy presented above.

The survey spectrum of the TiO_2_/rGO/SQ composite (Figure 4c) indicates that the composite contains Ti, O, C, and N, in agreement with the expected elemental composition of this material and suggests the successful incorporation of squaraine (SQ) into the TiO_2_/rGO catalyst. The O1s spectrum (Figure 4d) shows two peaks centered at 529.9 eV and 531 eV. The peak at 529.9 eV can be attributed to lattice oxygen (Ti-O-Ti) on the TiO_2_ framework, while the peak with binding energy 531 eV corresponds to surface hydroxyl groups and/or Ti-O-C species formed at the TiO_2_/rGO composite [42]. The high-resolution Ti 2p spectrum (Figure 4e) exhibited two well-defined peaks located at 458.7 and 464.5 eV, which can be assigned to Ti 2p3/2 and Ti 2p1/2 of Ti^4+^ in the TiO_2_ lattice, respectively [41,43]. The N 1s spectrum (Figure 4f) shows a characteristic peak centered at 399 eV, attributed to the nitrogen atoms of the squaraine dye SQ, and confirms the successful incorporation of SQ molecules into the composite [43].

The X-ray diffraction (XRD) pattern of TiO_2_/rGO/SQ (Figure 5) shows the reflections characteristic of anatase TiO_2_: (101) at 25.3°, (004) at 37.8°, and (200) at 48.0°, as well as the weak (103)/(112) shoulders in the 37°–38.6° (JCPDS 21-1272). The reflection of rGO (002) at ≈24° overlaps with the intense anatase (101) reflection and cannot be distinguished separately. The anatase crystallite size estimated from the broadening of the (101) reflection using the Scherrer equation is 14 nm.

Pure SQ is crystalline, exhibiting intense reflections at 2θ = 8.1°, 12.8°, 18.7°, 21.0°, and 21.8°. Weak reflection features at 12.9°, 18.7°, 21.1°, and 21.8° are also observed in the diffraction pattern of TiO_2_/rGO/SQ (Figure 5), indicating that a part of the dye is retained in the composite as small crystalline aggregates, while the crystal structure of the TiO_2_ is unaffected by the dye deposition. The preservation of the characteristic anatase reflections indicates that the crystalline structure of TiO_2_ is retained after SQ deposition.

The morphology of the composites was examined by SEM under identical imaging conditions (Figure 6). TiO_2_/rGO consists of agglomerates of near-spherical TiO_2_ nanoparticles with a primary particle size of approximately 20–40 nm, forming a porous network with interparticle voids. The rGO sheets are largely covered by these agglomerates.

In TiO_2_/rGO/SQ (Figure 6), extended rGO sheets with relatively smooth surfaces and wrinkled, folded edges are clearly exposed, while the TiO_2_ nanoparticles are distributed over them as discrete clusters. The primary particle size of TiO_2_ appears to remain essentially unchanged. SQ is not visible or distinguishable in the SEM images because it forms a molecular layer on the surface, which is below the resolution of the method. The presence of the SQ in the composite is supported by FT-IR and XPS results.

The optical properties of TiO_2_/rGO and TiO_2_/rGO/SQ were investigated by UV–Vis diffuse reflectance spectroscopy. The diffuse reflectance data were transformed using the Kubelka–Munk function, F(R). As shown in Figure 7, incorporation of SQ leads to a pronounced enhancement of the optical response over a broad region of the visible spectrum, with distinct absorption features observed approximately in the 500–720 nm range. These features are associated with the contribution of the squaraine sensitizer and indicate improved light absorption by the TiO_2_/rGO composite. This observation supports the proposed role of SQ as the primary visible-light sensitizer in the photocatalytic process.

### 3.2. Photocatalytic Study of Prepared Composites

The absorption spectrum of the synthesized SQ dye in ethanol is shown in Figure 8. Typically, for the squaraines, the compound SQ strongly absorbs light in the red range of the visible. The photocatalytic decolorization efficiency of the synthesized TiO_2_/rGO/SQ and TiO_2_/rGO sample composites was determined toward the decomposition of the dyes RhB and FL. The decolorization of these dyes was evaluated through monitoring the changes in their characteristic absorption bands peaked at 488 nm for FL and at 552 nm for RhB (Figure 8).

To quantitatively assess the intrinsic absorption properties of FL and RhB, their molar absorption coefficients were experimentally determined using calibration plots based on the Beer-Lambert law. For FL, a good linear relationship between absorbance and concentration was established in the studied range of 1 × 10^−6^ to 1 × 10^−5^ M at λ_max_ = 489 nm, yielding a molar absorption coefficient of 2.38 × 10^4^ L mol^−1^ cm^−1^ (R^2^ = 0.9979). For RhB, the calibration plot at λ_max_ = 550 nm demonstrated excellent linearity within the concentration range of 1 × 10^−7^ to 1 × 10^−5^ M (R^2^ = 0.9998), with a molar absorption coefficient of 9.31 × 10^4^ L mol^−1^ cm^−1^. The significantly different molar absorption coefficient values indicate that the absolute absorbance values for FL and RhB are not directly comparable. Therefore, the results of the photocatalytic studies were evaluated using the normalized concentration (C/C_0_) rather than absolute absorbance values. The decolorization efficiency was determined from the absorbance data using the Beer-Lambert law:

$$\mathrm{Degree}\;\mathrm{of}\;\mathrm{decolorization}\;(\%)\;=\;\left(\frac{C_{0}-C}{C_{0}}\right)\times \;100,$$

where C_0_ and C are the initial concentration before illumination and the residual concentration of the dye solution after illumination for a selected time interval, respectively.

The photocatalytic activity of TiO_2_/rGO/SQ and TiO_2_/rGO toward decolorization of the dyes RhB and FL upon visible-light illumination is presented in Figure 9. After 180 min of irradiation, 98% decolorization of FL and 86% decolorization of RhB were achieved with TiO_2_/rGO/SQ (Table 2), demonstrating a high photocatalytic activity under visible-light irradiation. The apparent rate constants, determined over the full irradiation period, were 2.8-fold (FL) and 1.9-fold (RhB) higher upon SQ sensitization (Table 2). The decolorization of both dyes over the investigated composites followed apparent pseudo-first-order kinetics, as evidenced by the linear dependence of ln(C_0_/C) on irradiation time.

Since some organic dyes are susceptible to photolysis under visible-light irradiation, control tests were carried out to inspect the self-degradation of both dyes, RhB and FL, under the conditions of the present experiment. Upon irradiation with visible light for 30 min, less than 1% of FL underwent photolysis, while RhB exhibited 2.8% self-degradation over the same period; i.e., the self-degradation of both dyes is relatively low.

The photocatalytic performance of the TiO_2_/rGO composite was enhanced by photosensitization with the synthesized squaraine dye (SQ). Unlike squaraine dyes containing carboxylic acid anchoring groups [19], the SQ dye 3-oxo-4-[(1,3,3-trimethyl-3H-indol-1-ium-2-yl)methylene]-2-[(1,3,3-trimethylindolin-2-ylidene)methyl]cyclobut-1-enolate (inner salt) does not possess functional groups capable of forming strong covalent Ti–O–C linkages with the TiO_2_ surface. Nevertheless, adsorption of the SQ molecules on TiO_2_ is expected to occur through coordination of the oxygen atoms of the squaraine core with coordinately unsaturated Ti(IV) surface sites. The enolate oxygen, which carries a substantial negative charge owing to the zwitterionic character of the molecule, is expected to act as the primary coordination site toward Lewis acidic Ti(IV) centers. In addition, the adjacent carbonyl oxygen may also participate in the interaction, giving rise to monodentate or, depending on the adsorption geometry, weak bidentate coordination. Such Lewis acid–base interactions are expected to stabilize the dye on the TiO_2_ surface, although with lower binding strength than that achieved by carboxylate anchoring groups. Besides coordination bonding, weaker intermolecular interactions, including electrostatic interactions, hydrogen bonding with surface hydroxyl groups, and π–π interactions between neighboring dye molecules, may further contribute to stabilization of the adsorbed layer. Consequently, the adsorption strength and surface coverage are expected to be lower than those reported for carboxyl-functionalized squaraine dyes, but still sufficient to enable visible-light sensitization and electron injection into the TiO_2_ conduction band. Upon visible-light excitation, the excited-state electron of the adsorbed squaraine dye can be injected into the conduction band of TiO_2_, leaving the dye in its oxidized state. The injected electron subsequently participates in the reduction of dissolved oxygen to generate reactive oxygen species, while the oxidized dye is regenerated through electron transfer from the adsorbed organic substrate or intermediate species, thereby completing the photocatalytic cycle.

The enhanced photocatalytic performance of TiO_2_/rGO/SQ may arise from synergistic interactions among the individual components of the composite. The synergistic effect driven by specific interactions yields good results in the decolorization of the dyes RhB and FL under visible light irradiation, highlighting the potential of the TiO_2_/rGO/SQ composite for water purification applications.

The squaraine dye SQ possesses a stable, highly conjugated donor–acceptor–donor molecular structure and exhibits strong absorption in the visible and near-IR regions, enabling a possibility for enhancement of the photocatalytic activity of the TiO_2_/rGO composite. Some research groups have reported that the sensitizer dye (squaraine molecules) can be immobilized on the surface of rGO through π–π stacking interactions between the conjugated aromatic π-system of the dye and the π-electron-rich graphene basal plane [43]. The main function of the squaraine dye is to indirectly initiate the formation of O_2_•^−^ and, through subsequent reactions, potentially •OH. Upon irradiation with visible light, the photoexcited dye can transfer an electron to the conduction band of TiO_2_, generating a dye radical cation [44]. These electrons are captured by dissolved molecular oxygen, leading to the formation of superoxide radicals (O_2_•^−^) for the degradation of the pollutants. In many studies, such an electron-transfer pathway has been discussed for a variety of organic sensitizers, including squaraine dyes [45,46,47].

The injected electrons are efficiently transferred through the rGO sheets, which may facilitate electron transport and limit charge recombination. Subsequently, these electrons react with dissolved molecular oxygen to generate superoxide radicals (O_2_•^−^), which participate in the oxidative degradation of the organic pollutants. Similar electron-transfer pathways have been reported for a variety of dye-sensitized TiO_2_ photocatalysts, including systems employing squaraine dyes [46,47].

This mechanism is similar to that observed in porphyrin-sensitized rGO/TiO_2_ nanocomposite systems, in which the presence of the graphene component promotes the degradation of the contaminants and improves photocatalytic activity [46]. It is a highly efficient way to capture visible light and separate charge carriers to drive photocatalytic processes.

It is well established that the incorporation of an appropriate amount of rGO into TiO_2_ improves the visible-light photoresponse, enhances the charge-carrier separation in TiO_2_, and increases its photocatalytic efficiency. However, excessive graphene loading may result in the agglomeration of graphene sheets, shielding of the TiO_2_ surface, and reduced light-harvesting capability, ultimately leading to lower photocatalytic efficiency. Therefore, optimizing the TiO_2_/rGO ratio is essential for achieving high photocatalytic performance. The optimal ratio between the contents of the two components TiO_2_ and rGO, in rGO/TiO_2_ nanocomposite systems has been reported [48,49,50]. Previous studies have shown that TiO_2_/rGO nanocomposites containing 2–5 wt.% graphene oxide exhibit the highest photocatalytic activity toward the degradation of various organic pollutants under UV or visible-light irradiation. For this reason, in the present work, graphene oxide was added at a concentration of 2 wt.% to TiO_2_ to maximize photocatalytic efficiency for decolorization of dyes with visible light.

Both RhB and FL absorb in the visible range of the optical spectrum (Figure 8) and can act as photosensitizers. Upon excitation, electron transfer from the excited dye molecules to the conduction band of TiO_2_ may occur, promoting the generation of reactive oxygen species that contribute to dye degradation [51]. It should be noted that the dye acts both as the target pollutant to be degraded and as a photosensitizer that activates TiO_2_ upon irradiation with visible light. The mechanism of the dual-role “self-sensitization” process bypasses the limitation of only UV activation of TiO_2_, thus allowing TiO_2_ to utilize visible light [50,51,52]. Although self-sensitization of RhB and Fluorescein may contribute to the overall degradation process, the substantially higher photocatalytic activity of the TiO_2_/rGO/SQ composite compared with the TiO_2_/rGO composite demonstrates that the deliberate sensitization with SQ plays the dominant role in enhancing visible-light photocatalytic performance.

Vinu et al. employed Fluorescein and Eosin Y as visible-light sensitizers for the photocatalytic degradation of phenolic compounds over nano-TiO_2_ and demonstrated that the sensitizing dyes themselves undergo concomitant oxidative transformation through the indirect formation of highly reactive hydroxyl radicals (•OH) [53]. Similar observations have been reported by other researchers, indicating that Fluorescein-sensitized TiO_2_ can promote the degradation of organic pollutants under visible light, while the dye itself undergoes a concomitant oxidative transformation during the photocatalytic process [54].

In our case, it can be assumed that the excited dye molecules may transfer electrons into the rGO π-conjugation network, which facilitates their transfer to the conduction band of TiO_2_. This process favors the generation of reactive oxygen species, including superoxide radical anions (O_2_•^−^) and hydroxyl radicals (•OH), acting in parallel with the visible-light sensitization provided by the squaraine (SQ) dye. Generally, coupling TiO_2_ and rGO with dye sensitizers is a practical way to extend the activity of titania-based photocatalysts into the visible region. Our future research will focus on the development of sensitized photocatalytic systems that integrate deliberate sensitization with squaraine dyes and exploit the self-sensitization mechanism to degrade selected organic pollutants in aqueous solutions, improving degradation efficiency under visible-light irradiation.

The higher decolorization efficiency observed for FL compared with RhB can be due to differences in both the molecular structure and interfacial interactions of the molecules of the two dyes with the TiO_2_/rGO/SQ nanocomposite photocatalyst under the experimental conditions of our experiments. The oxygen-containing functional groups of FL may favor its interaction with the TiO_2_ nanosurface and facilitate efficient interfacial electron-transfer processes following visible-light excitation. In contrast, although RhB is also capable of photosensitizing TiO_2_ under visible-light illumination, its photocatalytic degradation involves competing oxidative pathways, including N-deethylation, which may contribute to the lower overall decolorization efficiency observed under the present conditions. Thus, the different responses of FL and RhB likely reflect differences in their molecular surface interactions and subsequent photochemical transformation pathways. In this context, the possible influence of some accompanying photochemical processes should also be mentioned, such as the generation of H_2_O_2_ during the photocatalytic degradation of both RhB and FL by visible light, reported in [55].

### 3.3. Reusability Study

The ability of photocatalysts to maintain their performance upon repeated use is important for their application. The reusability of the synthesized composites was evaluated over three consecutive photocatalytic cycles using the decolorization of the dyes RhB and FL as model reactions. At the end of each cycle, the photocatalysts were filtered, rinsed with deionized water, dried at 60 °C, and subsequently used again under identical experimental conditions. The results of the recyclability tests under visible-light irradiation are presented in Figure 10. The TiO_2_/rGO/SQ composite maintained high photocatalytic performance under visible-light irradiation, with the decolorization efficiency decreasing by only 8% for RhB and 6% for FL by the third cycle. This slight loss of activity could be related to the desorption of some SQ molecules from the catalyst surface. In contrast, the TiO_2_/rGO composite remained largely unchanged after repeated use, exhibiting excellent stability, with its decolorization efficiency decreasing by only 2–3% for both pollutants after the third cycle.

### 3.4. Proposed Photocatalytic Mechanism

According to the literature data, the photocatalytic transformation of RhB and FL upon visible-light irradiation over the TiO_2_/rGO/SQ photocatalyst can be represented by the following equations and is schematically illustrated in Figure 11:

SQ + *h*v → SQ* (1)

SQ* → SQ•^+^ + e^−^ (CB, TiO_2_) (2)

e^−^ (CB, TiO_2_) → e^−^ (rGO) (3)

e^−^ (rGO) + O_2_ → O_2_•^−^ (4)

O_2_•^−^ + H^+^ → HO_2_• (5)

2HO_2_• → H_2_O_2_ + O_2_ (6)

H_2_O_2_ + e^−^ → •OH + OH^−^ (7)

RhB/FL + *h*v → RhB*/FL* (8)

RhB*/FL* → RhB.^+^/FL.^+^ + e^−^ (CB, TiO_2_) (9)

O_2_•^−^/•OH + RhB/FL → reaction products (10)

Under visible light irradiation, the SQ molecules are promoted to the excited state SQ*, which can inject an electron into the conduction band of TiO_2_ (Equations (1) and (2)). The rGO accepts and transports the injected electrons, thereby suppressing charge recombination and promoting electron transfer to dissolved O_2_ to O_2_^−^ (Equations (3) and (4)). In aqueous medium, O_2_•^−^ can undergo protonation to HO_2_•, followed by subsequent reduction to H_2_O_2,_ affording hydroxyl radicals (Equations (5)–(7)). These reactive oxygen species can oxidize RhB and FL, leading to their degradation and decolorization upon exposure to visible light. The possible generation of such reactive oxygen species in squarylium dye-sensitized TiO_2_ photocatalysis has been reported in the literature. The formation of reactive oxygen species was demonstrated by EPR spectroscopy and peroxide assays for a squarylium cyanine dye-sensitized TiO_2_ system under visible-light irradiation [56]. The formation of H_2_O_2_ has also been confirmed during the visible-light degradation of RhB, FL, and squarylium dyes on TiO_2_ [55]. In addition, •OH formation on dye-sensitized TiO_2_ under visible-light irradiation has been directly detected using terephthalic acid fluorescence and EPR spectroscopy [57]. These literature findings support the proposed ROS-mediated pathway; however, the contribution of individual reactive species in the present TiO_2_/rGO/SQ system remains to be experimentally verified.

## 4. Conclusions

A TiO_2_/rGO/SQ composite photocatalytic system was successfully synthesized via a solvothermal method and used for photocatalytic decolorization of two organic dyes—Rhodamine B (RhB) and Fluorescein (FL). The photocatalytic reaction through TiO_2_/rGO/SQ was driven by photoexcitation of the component SQ in the composite, employed as a sensitizer. Sensitization with SQ effectively extended the photoresponse of the TiO_2_/rGO composite into the visible spectral range and significantly enhanced the photocatalytic activity of this nanocomposite under visible-light irradiation.

The synthesized TiO_2_/rGO/SQ composite photocatalyst exhibited excellent photocatalytic performance toward the decolorization of RhB and FL in aqueous solutions upon visible-light irradiation. Compared with the TiO_2_/rGO nanocomposite, the SQ-sensitized photocatalyst achieved 2.8-fold and 1.9-fold higher rate constants for FL and RhB, respectively, demonstrating the beneficial effect of SQ sensitization. The enhanced photocatalytic activity was attributed to the synergistic effects of visible-light absorption by the SQ dye, efficient charge separation and electron transport through rGO, and the generation of reactive oxygen species responsible for pollutant decolorization. Furthermore, the TiO_2_/rGO/SQ composite has retained the mesoporous structure by the incorporation of SQ and exhibited good stability during repeated photocatalytic cycles, indicating its potential for practical environmental applications. The strategy of combining semiconductor modification with graphene-based charge separation and squaraine dye sensitization provides an effective approach for designing visible-light-driven photocatalytic materials for environmental remediation.

## Figures and Tables

**Figure 1:**
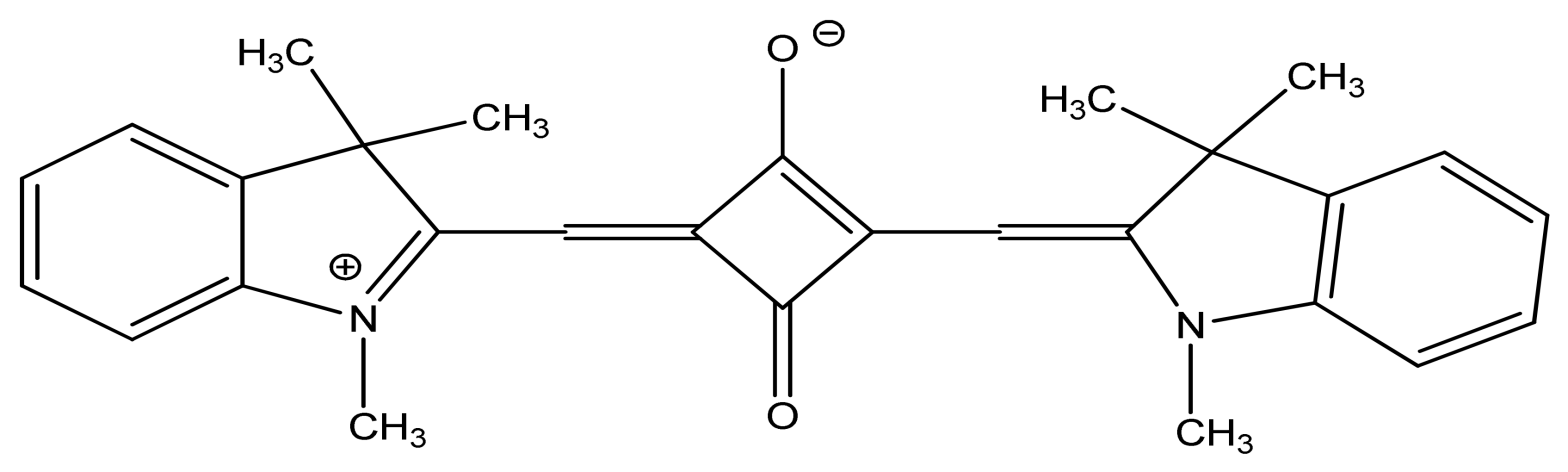
Molecular structure of the squaraine dye used as a sensitizer.

**Figure 2:**
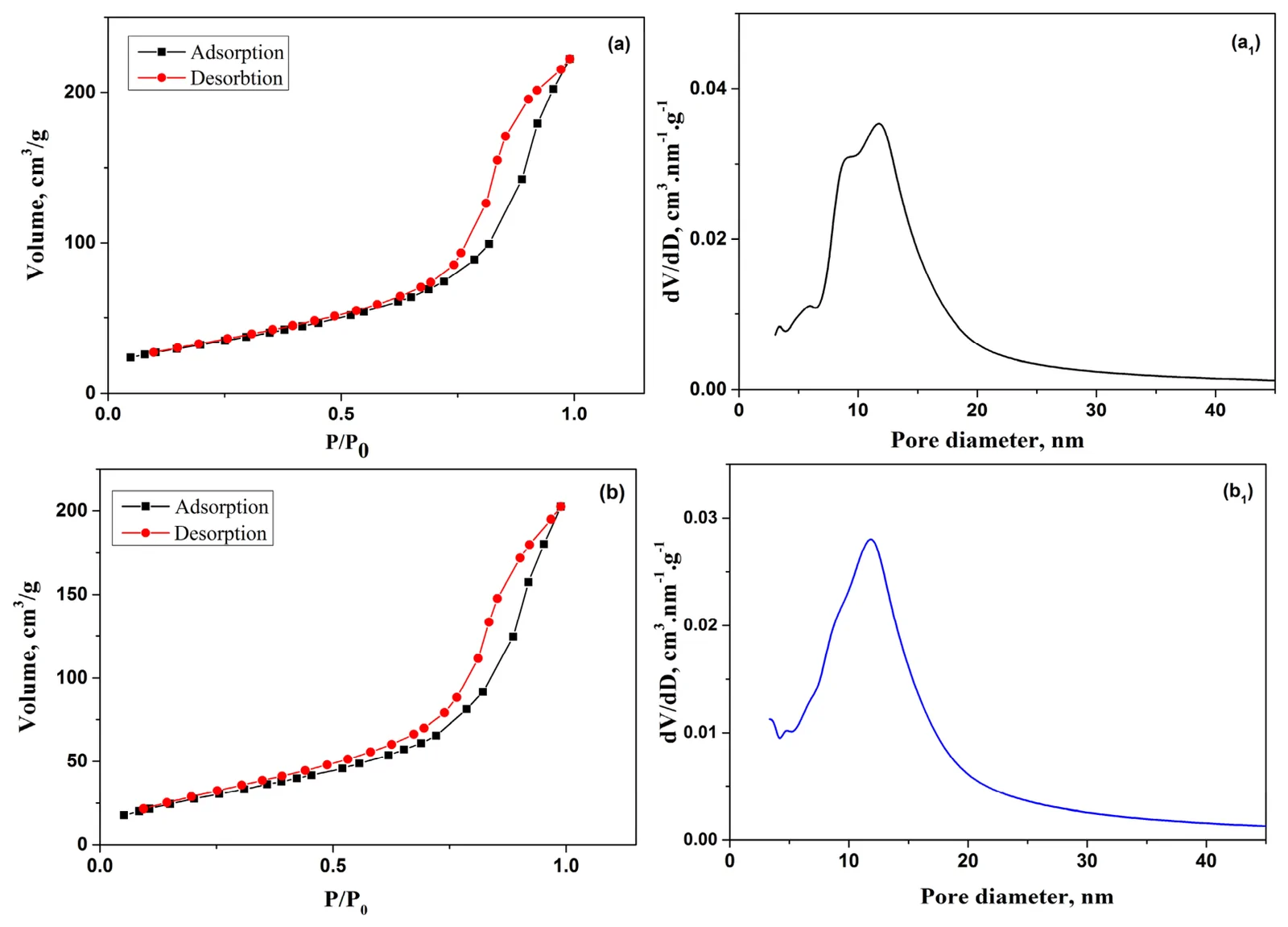
N_2_ adsorption-desorption isotherm and pore size distribution of (**a**,**a_1_**)—TiO_2_/rGO and (**b**,**b_1_**)—TiO_2_/rGO/SQ catalyst.

**Figure 3:**
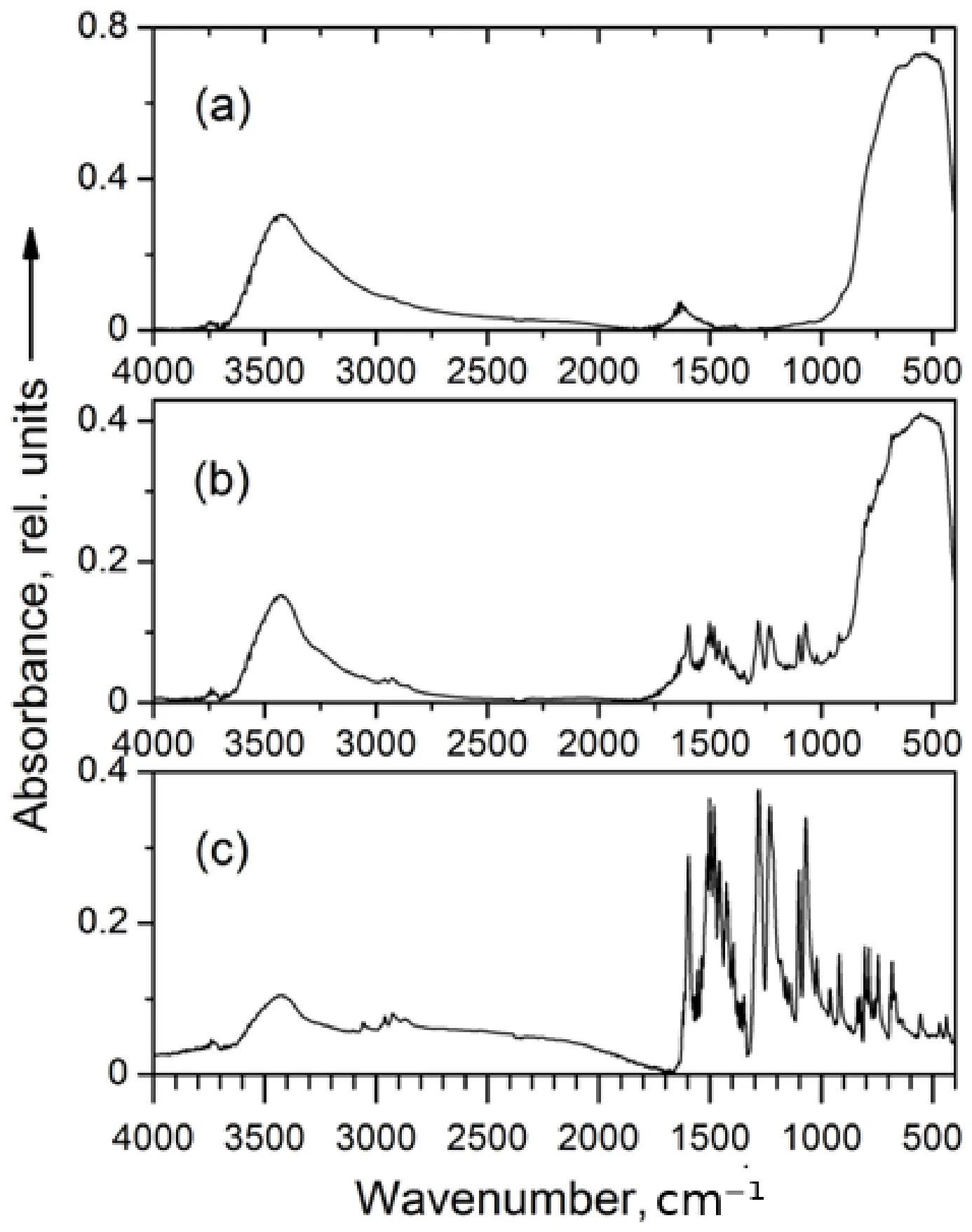
FT-IR spectra of TiO_2_/rGO (**a**); TiO_2_/rGO/SQ (**b**) and SQ (**c**).

**Figure 4:**
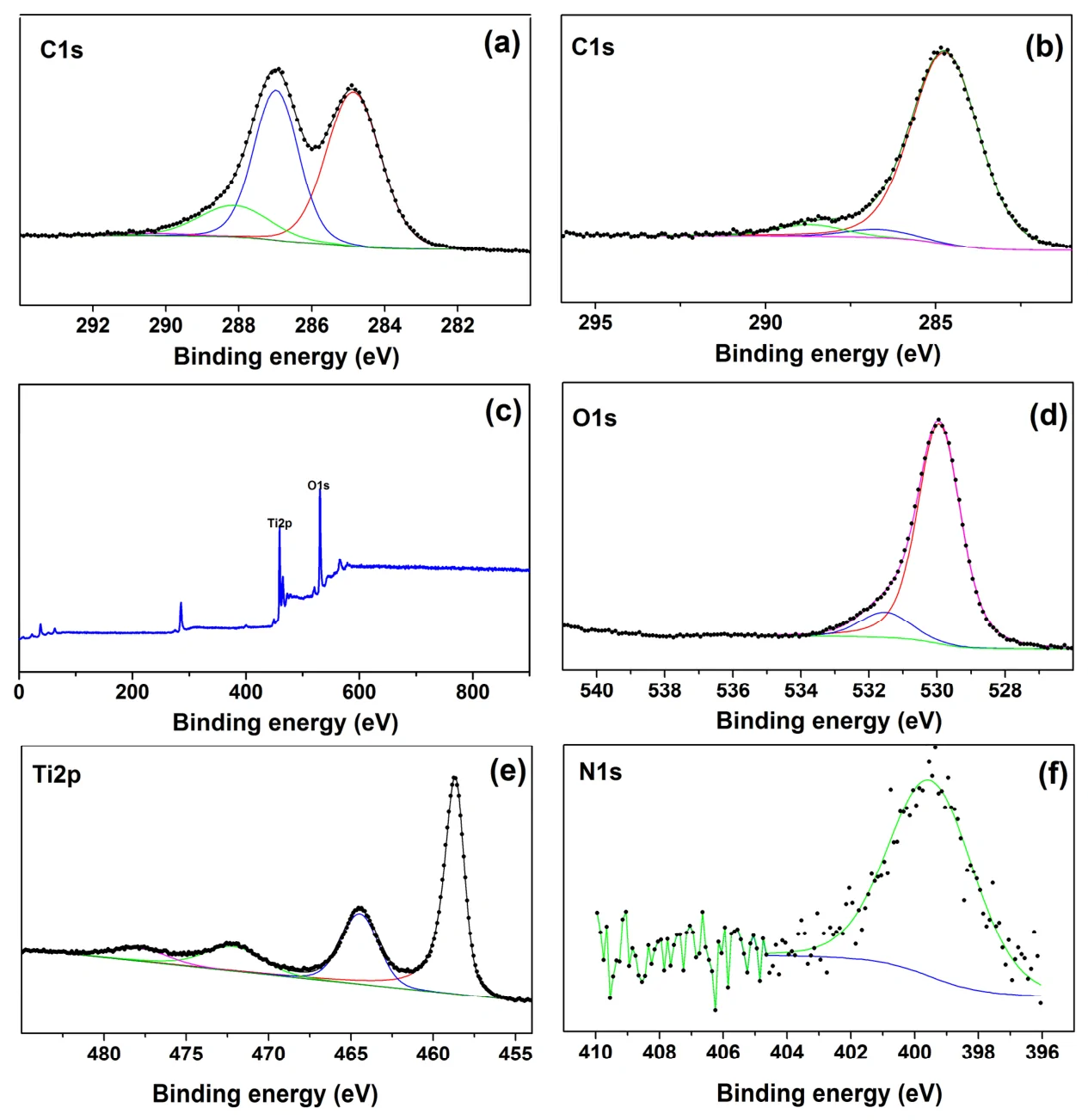
XPS spectra of C1s for graphene oxide (**a**) and TiO_2_/rGO/SQ (**b**); (**c**) survey scan; (**d**) O1s; (**e**) Ti2p; (**f**) N1s. Black dots represent the experimental data, while the colored lines represent the fitted components.

**Figure 5:**
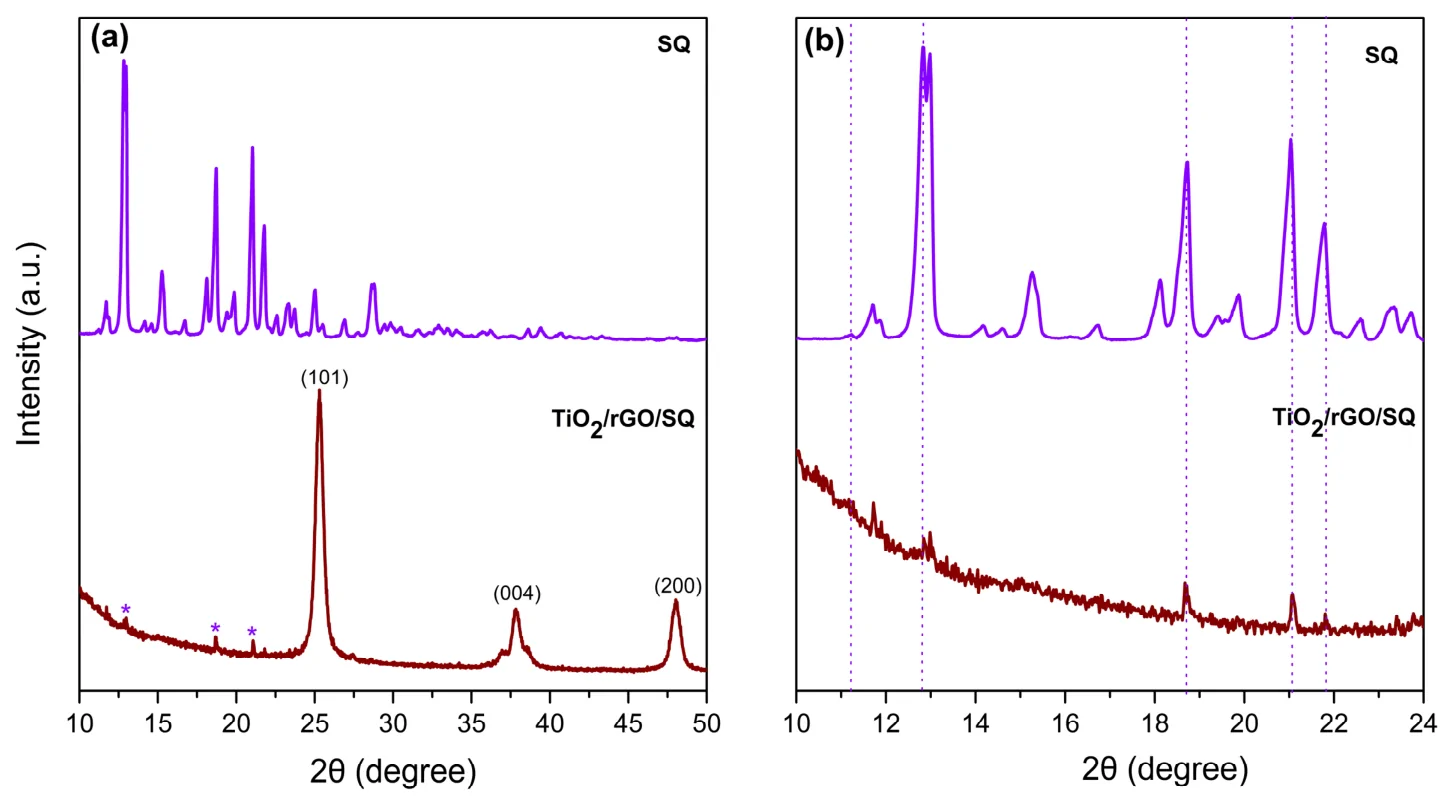
XRD patterns of TiO_2_/rGO/SQ and of pure SQ: (**a**) XRD patterns in the 2θ range of 10–50°; (**b**) enlarged view of the XRD patterns in the 2θ range of 10–24°. The dark-red and violet lines correspond to TiO_2_/rGO/SQ and pure SQ, respectively. The asterisks (*) indicate reflections attributed to SQ in the TiO_2_/rGO/SQ composite, while the vertical violet dotted lines indicate the positions of the corresponding reflections of pure SQ.

**Figure 6:**
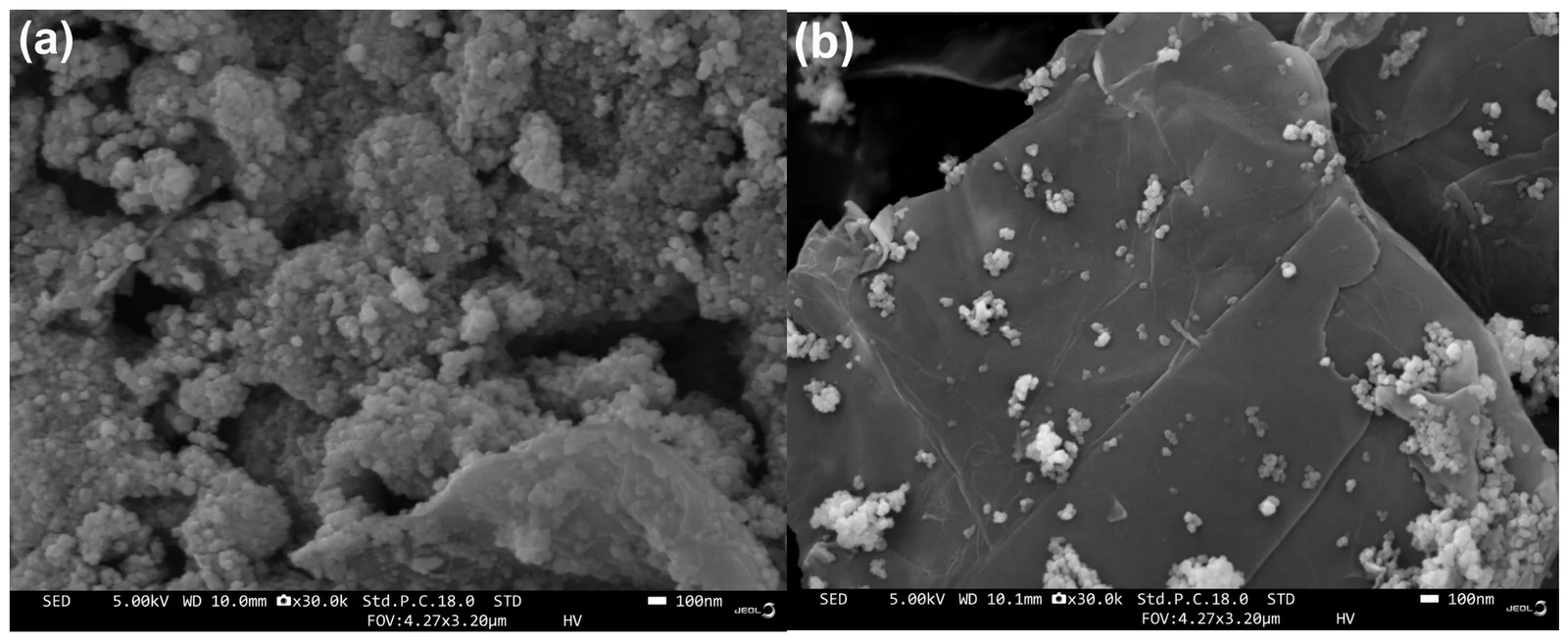
SEM images (secondary electron mode, 5 kV, ×30,000) of (**a**) TiO_2_/rGO and (**b**) TiO_2_/rGO/SQ. Scale bars: 100 nm.

**Figure 7:**
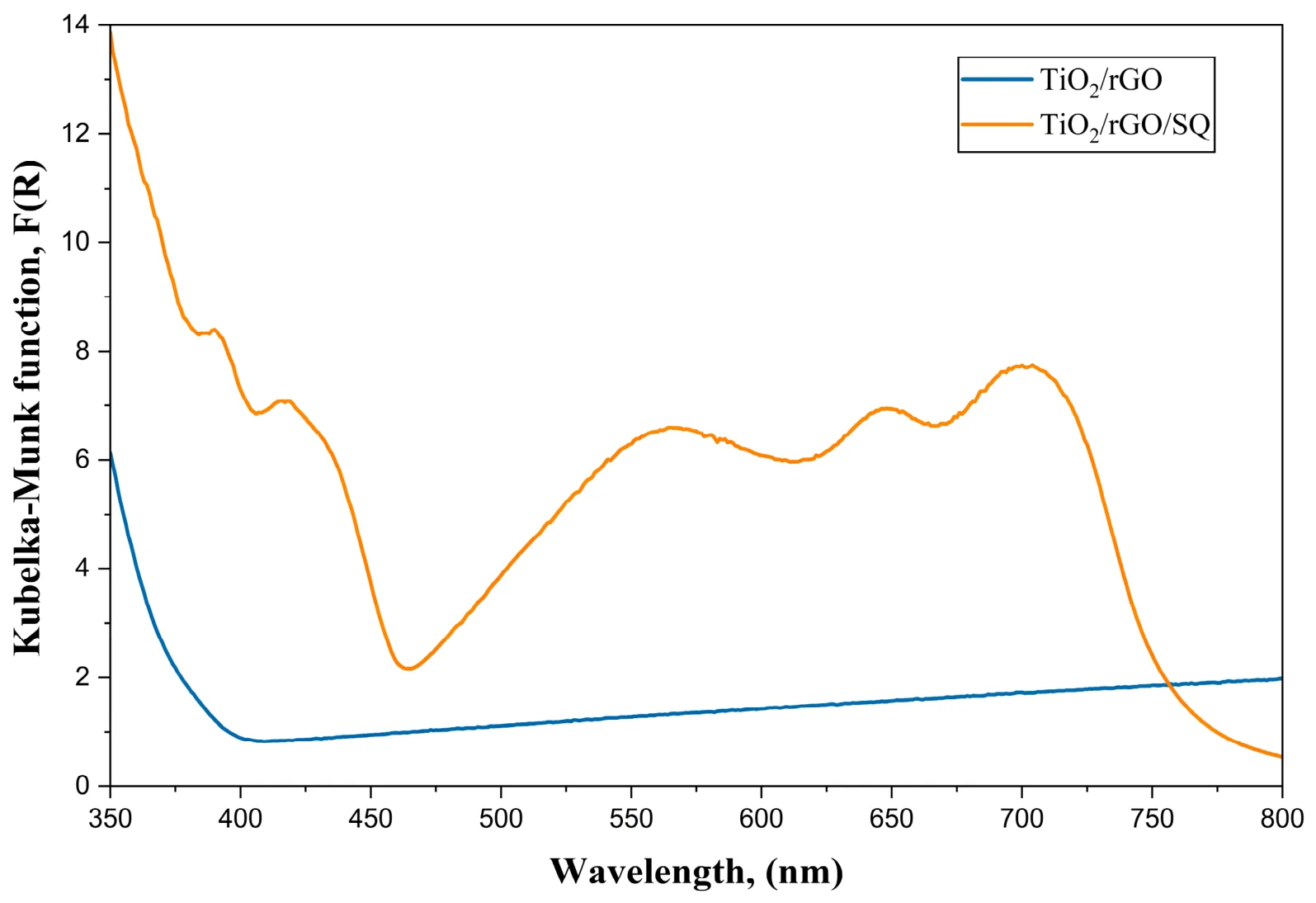
UV–Vis diffuse reflectance spectra of TiO_2_/rGO and TiO_2_/rGO/SQ expressed as the Kubelka–Munk function, F(R).

**Figure 8:**
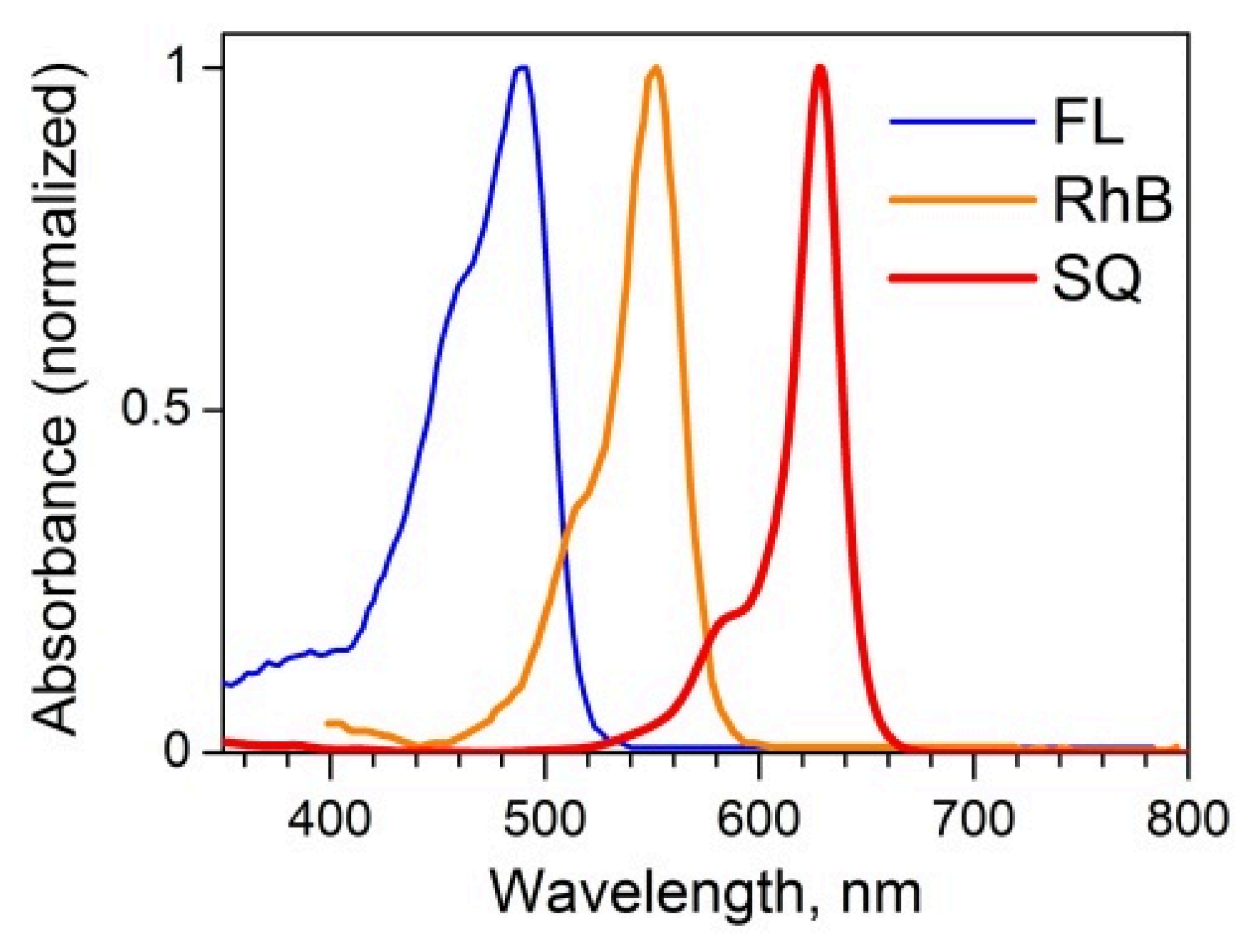
Absorption spectra of solutions (10^−5^ M) of FL and RhB in water, as well as SQ in ethanol.

**Figure 9:**
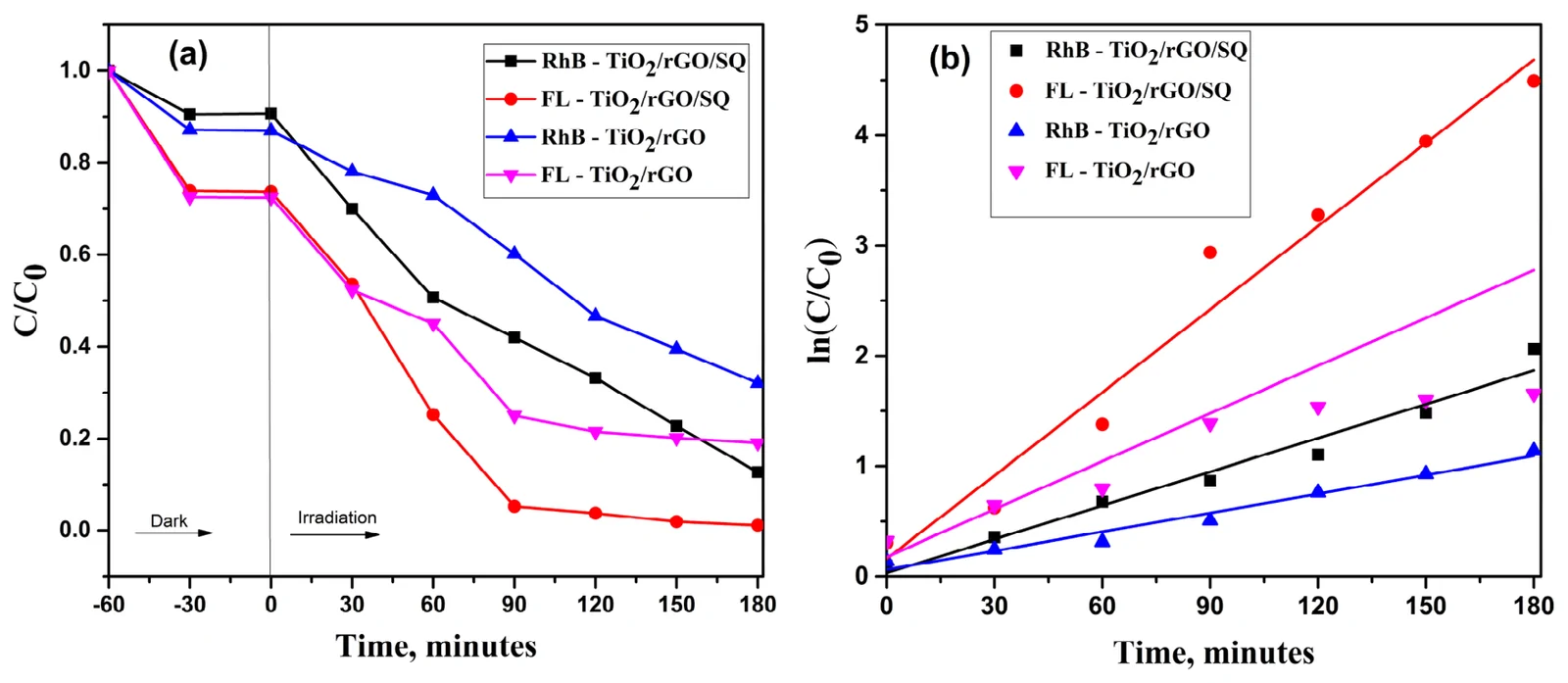
Concentration ratio C/C_0_ measured for the dyes RhB and FL as a function of the time of the visible-light illumination (**a**); kinetic curves of decolorization of RhB and FL (**b**).

**Figure 10:**
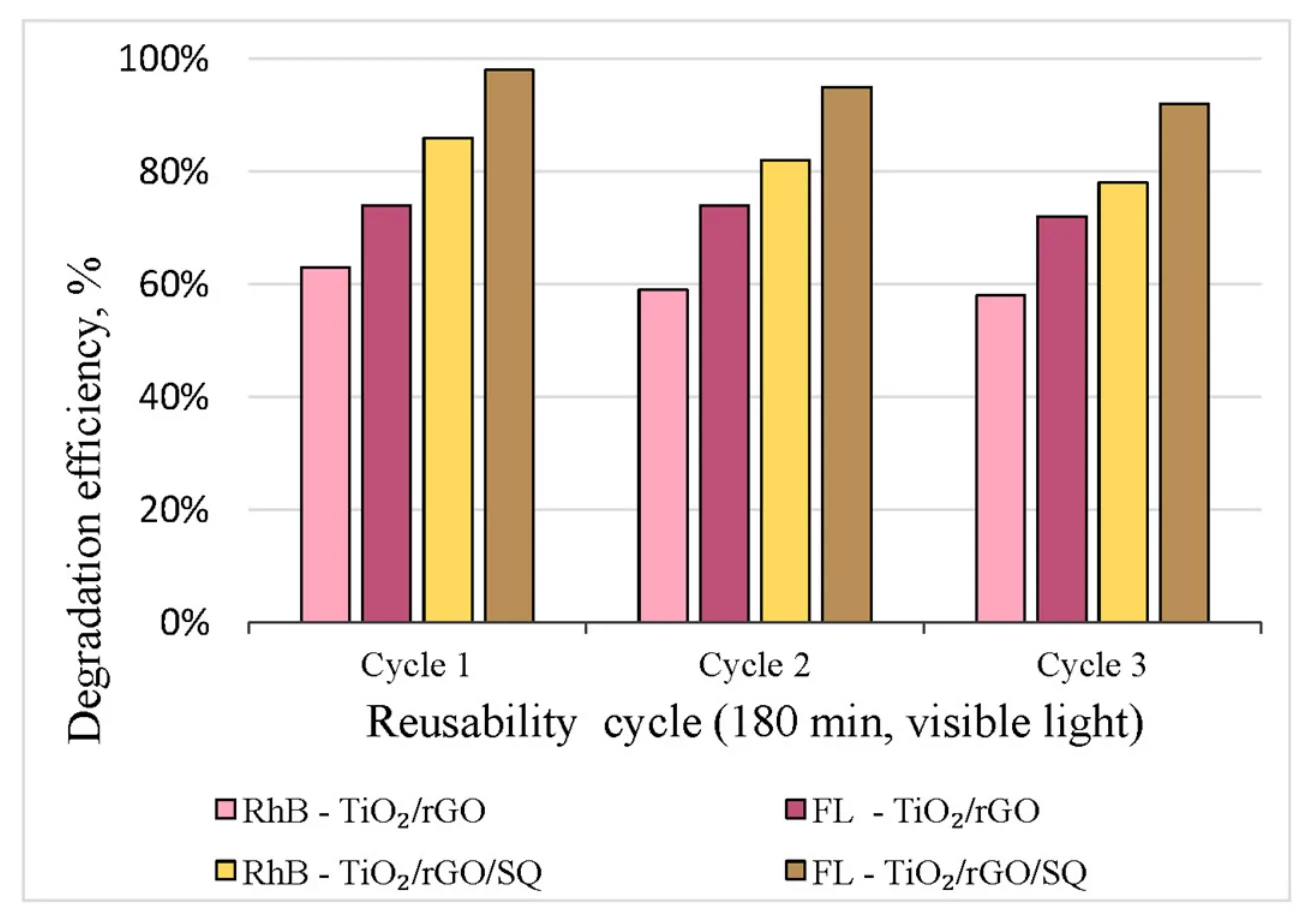
Reusability tests for the prepared composite photocatalysts (TiO_2_/rGO and TiO_2_/rGO/SQ), repeated 3 times, presenting the degree of RhB and FL dye decolorization.

**Figure 11:**
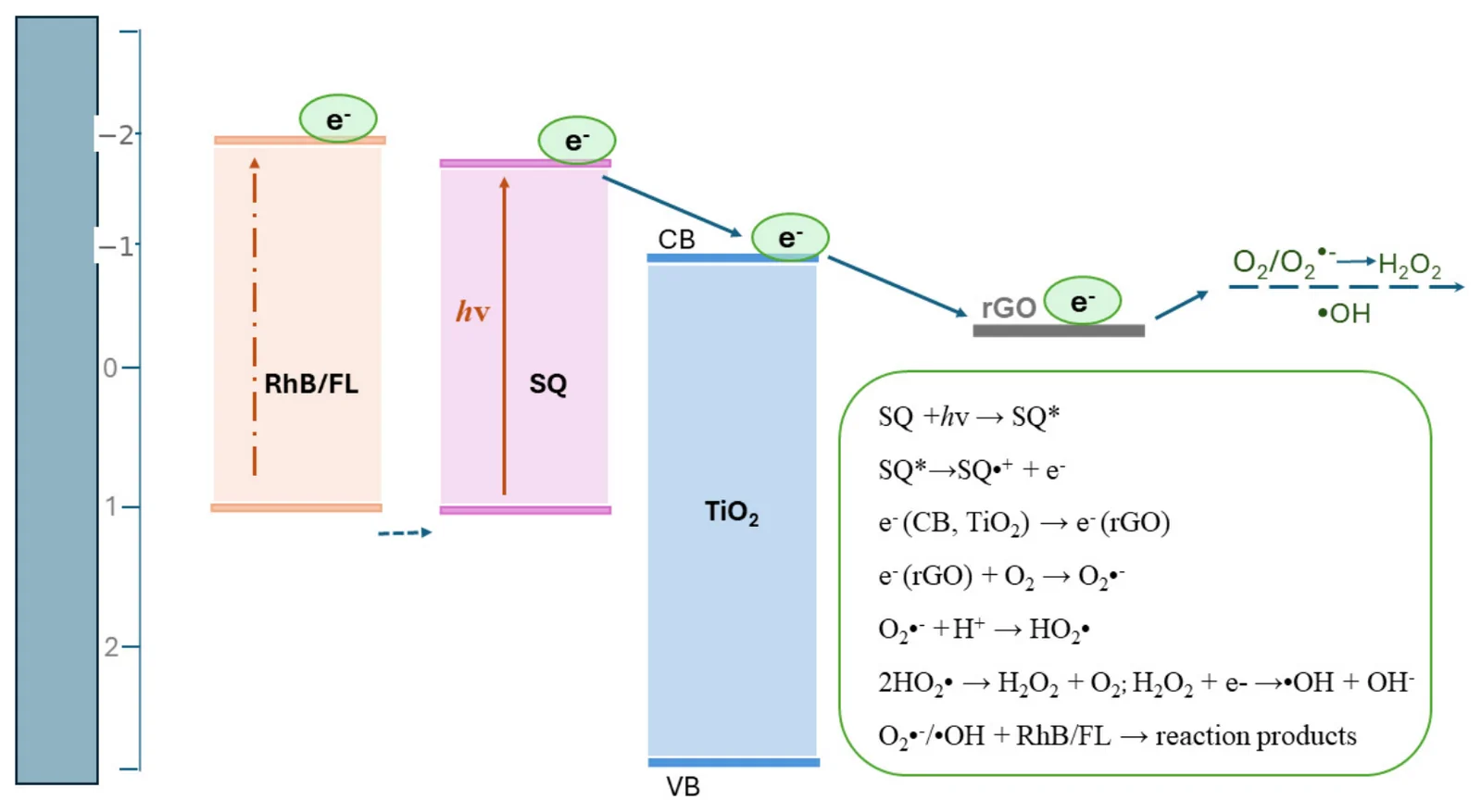
Schematic illustration of the proposed mechanism of decolorization of RhB and FL over TiO_2_/rGO/SQ composite. Solid arrows indicate photoexcitation, electron transfer, and subsequent reaction pathways, while dashed arrows indicate the photoexcitation and sensitization pathway of RhB/FL. The asterisk (*) denotes the photoexcited state.

**Table 1:** Textural properties determined by N_2_ adsorption isotherms of the studied composites: BET surface area (S_BET_), total pore volume (V_t_), and average pore size/diameter (D_av_).

Sample	S_BET_, m^2^/g	V_t_, cm^3^/g	D_av_, nm
TiO_2_/rGO	118 ± 2	0.35 ± 0.01	11.9 ± 0.3
TiO_2_/rGO/SQ	106 ± 2	0.31 ± 0.01	11.7 ± 0.3

**Table 2:** Decolorization efficiencies and apparent rate constants for the photocatalytic decolorization of Rhodamine B and Fluorescein over TiO_2_/rGO and TiO_2_/rGO/SQ under visible-light irradiation.

Catalyst	Dye	Decolorization (%)	K_app_ × 10^−3^, min^−1^	R^2^
TiO_2_/rGO	Rhodamine B	63	5.1	0.960
TiO_2_/rGO	Fluorescein	74	8.7	0.962
TiO_2_/rGO/SQ	Rhodamine B	86	9.7	0.967
TiO_2_/rGO/SQ	Fluorescein	98	24.1	0.967

## Data Availability

The original contributions presented in this study are included in the article. Further inquiries can be directed to the corresponding authors.

## Abbreviations

The following abbreviations are used in this manuscript:

RhB	Rhodamine B
FL	Fluorescein
SQ	Squaraine dye
rGO	Reduced graphene oxide
